# Dental age estimation: periodontal ligament visibility (PLV)—pattern recognition of a conclusive mandibular maturity marker related to the lower left third molar at the 18-year threshold

**DOI:** 10.1007/s00414-016-1468-3

**Published:** 2016-11-03

**Authors:** Victoria S. Lucas, Fraser McDonald, Manoharan Andiappan, Graham Roberts

**Affiliations:** King’s College London Dental Institute, London, UK

**Keywords:** Dental age estimation, 18-year threshold, Periodontal ligament visibility, PLV, Mandibular maturation markers, Probability estimates, Chronological age

## Abstract

The purpose of this study was to explore the applicability of periodontal ligament visibility (PLV) at the 18-year threshold. This mandibular maturity marker is graded into four separate age related stages, PLV-A, PLV-B, PLV-C, and PLV-D. These are discernible on a dental panoramic tomograph (DPT). The sample comprised a total of 2000 DPTs evenly divided into half yearly age bands from 16.00 to 25.99 years with 50 females and 50 males in each age band. It was found that PLV-A and PLV-B had minimum values below the 18-year threshold. PLV-C and PLV-D in females had minimum values of 18.08 and 18.58 years, respectively. In males, the minimum values for PLV-C was 18.10 years and PLV-D was 18.67 years. It was concluded that the presence of PLV-C or PLV-D indicates that a subject is over 18 years with a very high level of probability.

## Introduction

Recently, the DARLInG research group demonstrated that root pulp visibility (RPV) provides a strong indication that a subject at or near the 18-year threshold can be assigned to above the 18-year threshold with confidence (Lucas et al. 2016 submitted). This is an important threshold as increasing numbers of adult looking subjects seeking asylum in Europe and North America are claiming to be under 18 years. This has led to a number of publications aimed at using the development of the lower third molar as an indication of mature status. The most common age threshold is 18 years as this, in legal terms, is the defining age between child and adult status [1].

At the 18-year threshold, the third molar is the only tooth that has not completed development. The effective use of the lower third molar to determine whether or not a subject is over 18 years was first described by the American Board of Forensic Odontology (ABFO) [2]. This was also the first time that the eight-stage system of tooth development stages (A–H) devised by the Anglo-Canadian research team at the Institute of Child Health in London [3] was used for forensic age estimation. An important aspect of the ABFO study was the reporting of summary statistics including the mean ($\overline{x}$) and standard deviation (sd) which were then converted to probability values. Thus, it was possible, for the first time, to easily tabulate the probability that the client was at least 18 years old using, in particular, stage H which is the final stage of development. The application of the principle of probability estimates has since been applied at the age of criminal responsibility in the UK [4, 5]. This proved to be a highly reliable method of assigning a child to the correct position above or below the 10-year threshold. The usual approach when assigning a subject to below or above the 18-year threshold is to estimate the cumulative probability with the cutoff at 18 years of age. This is usually based on calculations using the normal distribution [2, 6]. However, it has been shown recently, using the gold standard of chronological age as a comparator, that in the age range 17.5 to 19.5 years, there is a high risk of an incorrect assignment [7].

This raises concerns that a large proportion of children will be treated as adults, and alternatively, a large proportion of adults will be treated as children. This is inappropriate and lends support to the view that when assigning a subject to below or above the 18 threshold, it is important to be sure that the assignment has a very high level of probability. It was concluded that “Completed root growth in wisdom teeth is observable at ages less than 18 years.” [8].

An important statement as in the UK the assignment for a subject to over (or under) 18 years is based on the balance of probability that is to say greater (or less than) 50 %. In the USA, this is known as the preponderance of the evidence [9].

Other human biological growth markers (HBGM) assist in this process. One of these is the relative visibility of the periodontal ligament as seen on dental panoramic tomographs (DPT) [10]. A four-stage system was devised [10] whereby the decrease in periodontal ligament visibility (PLV) with increasing age demonstrated the potential to show that a subject is below or above the 18-year threshold. The importance of this is that using the data derived in that paper [10], it might be possible to determine whether or not an individual subject is over 18 years old from a single DPT. It is important to note that this applies only when the lower third molar has completed growth i.e., stage H of the eight-stage system [3]. Thus, the use of PLV may assist in determining whether or not a subject without birth records is above or below the 18-year threshold.

This report is aimed at using the mandibular maturity marker of PLV to determine whether this specific HBGM is a reliable indicator that a subject is over 18 years old in a UK-Caucasian group of females and males.

## Materials and methods

Ethical approval for this study was granted by the Oxford C National Research Ethics Committee—Number 12/SC/0029.

The radiographical archives of Guy’s and St Thomas’ NHS Trust were searched for subjects whose registration details provided the date of birth, gender, and ethnicity. From the radiographic database, subjects were selected to provide 50 females and 50 males in six monthly age bands from 16.00 to 25.99 years. These are the same radiographs that were used to determine the reliability of the final stage of root growth of third molars in assigning subjects to below or above the 18-year threshold [7]. The assessors are experienced in viewing DPTs and categorising the TDSs [3]. In addition as the PLV is a relatively new HBGM, the two assessors received extensive training and validation prior to the onset of the main part of the investigation. All radiographs were examined in random order.

To manage data, separate Microsoft Excel worksheets for PLV were created by applying a cascade of filters containing the data for the 2000 cases used in the study.

1. The full data set was first partitioned by applying a filter to identify female and male subjects.
2. All subjects with completed root growth, the final stage tooth development, designated stage H in the eight-stage system of tooth development stages [3], were retained in the spreadsheet.
3. Following this, a further filter was applied to isolate the data for PLV-A, PLV-B, PLV-C, and PLV-D (Fig. 1). These schematic drawings and radiographic examples were used to assist the assessor in placing individual cases into the appropriate category. It was impossible to match the radiographic appearance of PLV as originally described [10]. The approach was modified so that the overall appearance of PLV with the approximate area of periodontal ligament for both mesial and distal roots was estimated and assigned to one of the categories. This decision to describe the periodontal ligament visibility of LL8H as an approximation of the percentage visibility was pragmatic, and after some training and experience with the concept, it was easy to identify the four categories described (Fig. 1). It was considered appropriate to rename the categories as A, B, C, and D so that the impression is not given that the differences between 0 and 1, 1 and 2, 2 and 3, and 3 and 4 were not whole numbers thus potentially giving the false indication that these categories were on a simple number scale [11]. For this reason, it is appropriate to use upper case letters A, B, C, and D to indicate each category. This is similar to the scheme used by the Anglo-Canadian research team but applied to PLV in an ordinal manner [12].

**Fig. 1:**
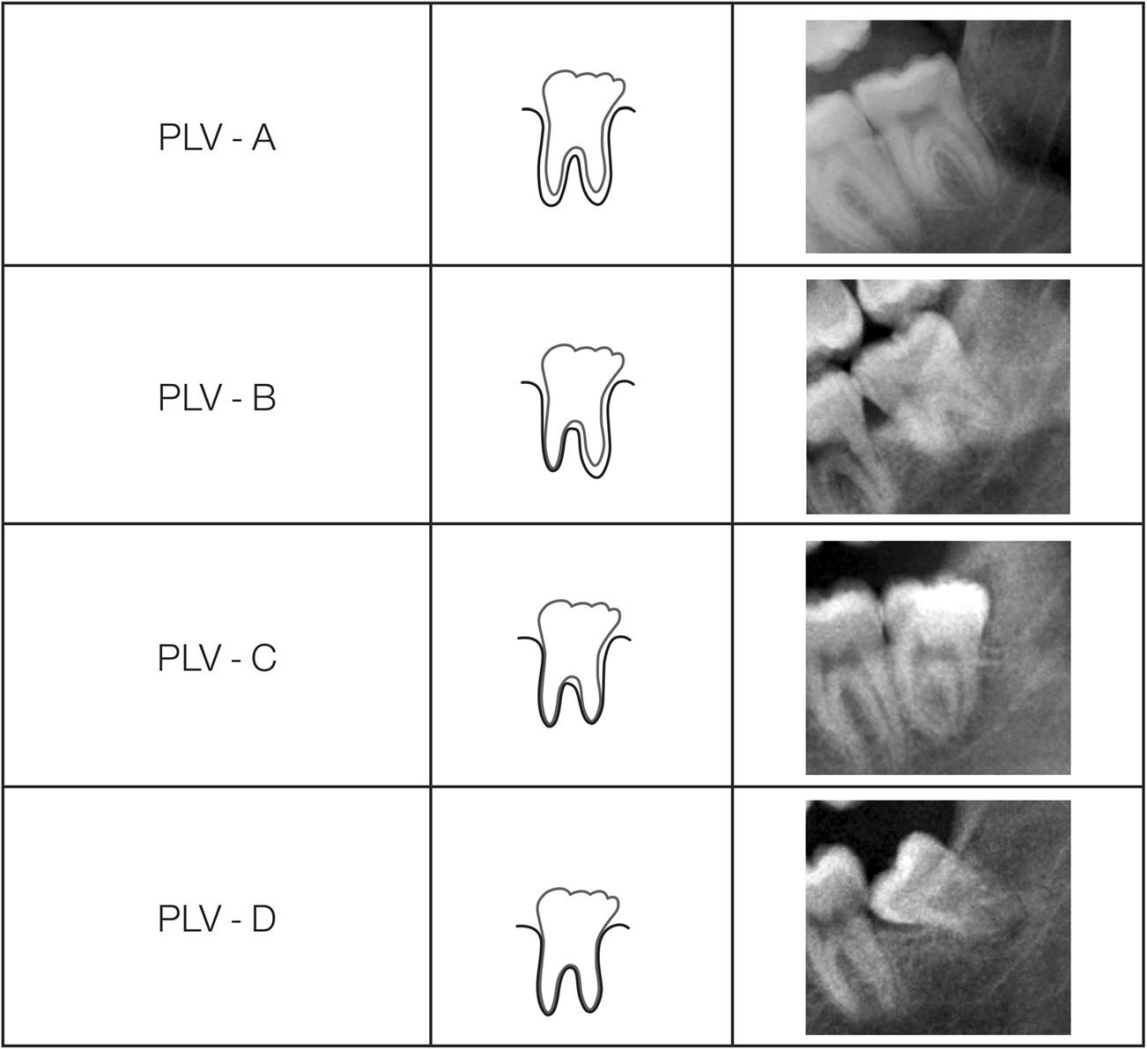
Schematic representations of the four grades of periodontal ligament visibility with examples of the radiographic representation of each RPV grade. Redrawn after Olze et al. 2010 [10], the appearances indicated in the schematic representations rarely appear with such a precise architecture. The diagrams represent the amount of visible periodontal ligament on the dental panoramic tomograph which is lost taken across the whole of the visible area of the mesial and distal roots combined and expressed as the area of the periodontal ligament that is no longer visible. This is essentially a form of pattern recognition. PLV-A = 100 % of periodontal ligament visible; PLV-B = 75 to 50 % of periodontal; ligament visible; PLV-C = 50 to 25 % of periodontal ligament visible; PLV-D = 0 % of periodontal ligament visible. It is helpful to enlarge the image using the computer software when making the assessments
4. PLV-A is assigned when 100 % of the periodontal ligament around the lower left third molar is visible.
  PLV-B is assigned when 75 to 50 % of the periodontal ligament is visible. This is when the pattern of PLV across both mesial and distal roots is mentally summated to be between 75 and 50 %.
  PLV-C is assigned when 50 to 25 % of the periodontal ligament of the lower left third molar is visible when summated across the mesial and distal roots.
  PLV-D is assigned when 0 % (or close to it) of the periodontal ligament of the lower left third molar is discernible i.e. 100% of the periodontal ligament of the lower left third molar has disappeared.
  This data was subjected to the Shapiro Wilk Test to test for normality [13].
5. The data for chronological age of each category of PLV were then ranked from the oldest at the top to the youngest at the bottom of the column, and the percentile for each rank was estimated using the in-built functions of Microsoft Excel. The ranked data are then visually scanned from the oldest down to the point immediately above 18.00 years. The rank for the value immediately above 18.00 indicates the probability that the value identified is above 18.00 years.
6. The summary statistics for each of the grades of PLV-A, PLV-B, PLV-C, and PLV-D was calculated and displayed in tabular form. This includes the mean ($\overline{x}$), standard deviation (sd), the minimum (0 %-ile), the 25th%-ile, the median (50th%-ile or median), the 75th%-ile, and the maximum (100th%-ile) and also includes the probability that a subject with a PLV feature is over 18 years. In addition, the probability distribution for the censored data of the final stage of development of the lower left third permanent molar (LL8H) for both females and males was plotted as a background setting for the data array for each of the PLV stages which are superimposed on the plot along with a vertical line to indicate the 18-year threshold.

### Repeatability

The within observer agreement (WOA) and between observer agreement (BOA) for the grades of PLV was assessed using Cohen’s Kappa [14].

Dental panoramic tomographs from 1000 females and 1000 males evenly distributed across the age ranges specified were examined. The principle investigator has been conducting DAE studies for over 10 years and has considerable experience in using the eight category system of tooth development stages.

## Results

### Repeatability

The repeatability of the categorical assessments for the periodontal ligament visibility for the WOA was 96.00 % agreement which gives a Kappa value of 0.9689. For the BOA, there was 97.97 % agreement which gives a Kappa value of 0.9690. These values are considered to be almost perfect [13].

### Summary statistics

The details of the data for each of the PLV grades are shown in Table 1. For both females and males, it can be seen that the *minimum* values for PLV-Cf, PLV-Df, PLV-Cm, and PLV-Dm return a value that is slightly above the 18-year threshold (the f suffix signifies female, the m suffix signifies male).

**Table 1:** Summary data for each of the grades of periodontal ligament visibility (PLV). f = female, m = male UK Caucasian data

	n-tds	$\overline{x}$ -tds	sd-tds	min-tds	25th%ile	50th%ile	75th%ile	max-tds	18-year threshold	*p* < 18	*p* > 18
Females											
PLV-Af	8	19.57	1.83	16.33	18.23	20.28	20.60	22.06	14.20 %	0.142	0.858
PLV-Bf	202	21.25	2.16	16.17	19.80	21.21	22.61	25.83	5.90 %	0.059	0.941
PLV-Cf	277	22.96	1.95	18.08	21.43	23.36	24.47	25.95	*0.00 %*	*0.000*	*1.000*
PLV-Df	54	23.86	1.79	18.58	22.66	24.33	25.39	25.99	*0.00 %*	*0.000*	*1.000*
Males											
PLV-Am	12	20.32	1.61	17.69	19.58	20.27	21.48	22.80	9.00 %	0.090	0.910
PLV-Bm	151	21.17	2.13	17.62	19.48	20.85	22.68	25.43	2.60 %	0.026	0.974
PLV-Cm	308	22.49	2.11	18.10	20.86	22.63	24.22	25.43	*0.00 %*	*0.000*	*1.000*
PLV-Dm	87	23.37	1.85	18.67	22.29	23.61	24.94	25.93	*0.00 %*	*0.000*	*1.000*

n-tds indicates the tooth development stages for the subgroup (PLV-A etc.) as distinct from *N* which refers to the full sample of 1000 females and 1000 males. The numbers in italics indicate a probability of being under or over 18-years as 0.000 [0%] and 1.000 [100% certainty]

**Fig. 2:**
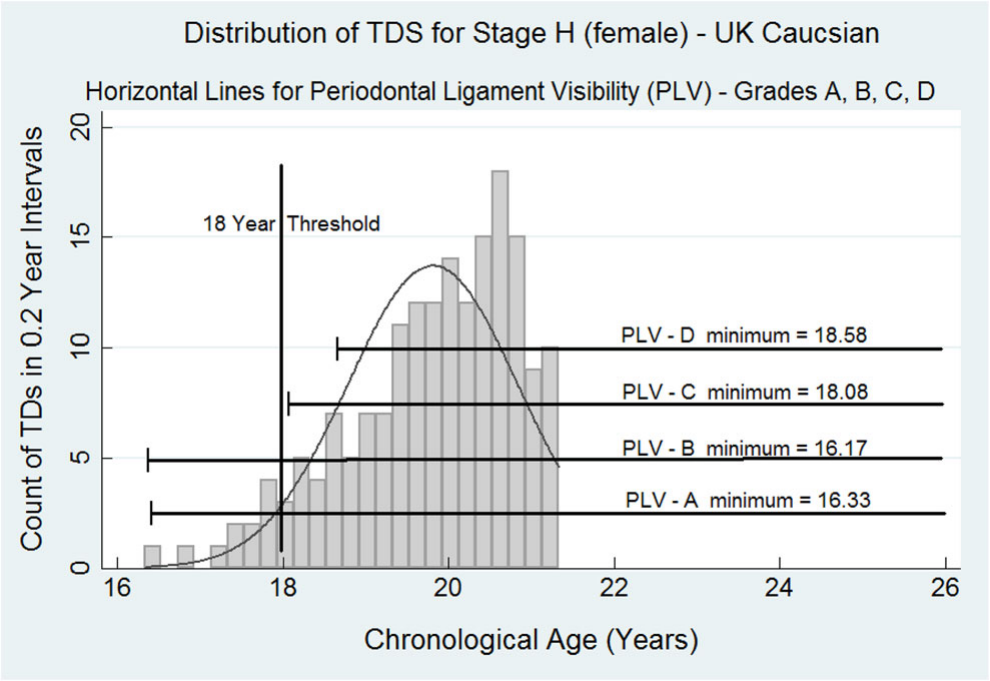
The histograms represent the probability distribution for the ages of attainment for tooth development stage H (the final stage of tooth development) for the lower third molar on the females. The data are censored at approximately 21.7 years for females. Superimposed are the *horizontal data bars* for PLV stages A, B, C, and D. The full summary data for these PLV stages in both females and males are shown in Table 1. The probability distribution curve for LL8H f and m, respectively, is present as a background to the *horizontal lines* encompassing the data for PLV-A, PLV-B, PLV-C, and PLV-D. Minimal data has been entered on the graphs to avoid clutter

**Fig. 3:**
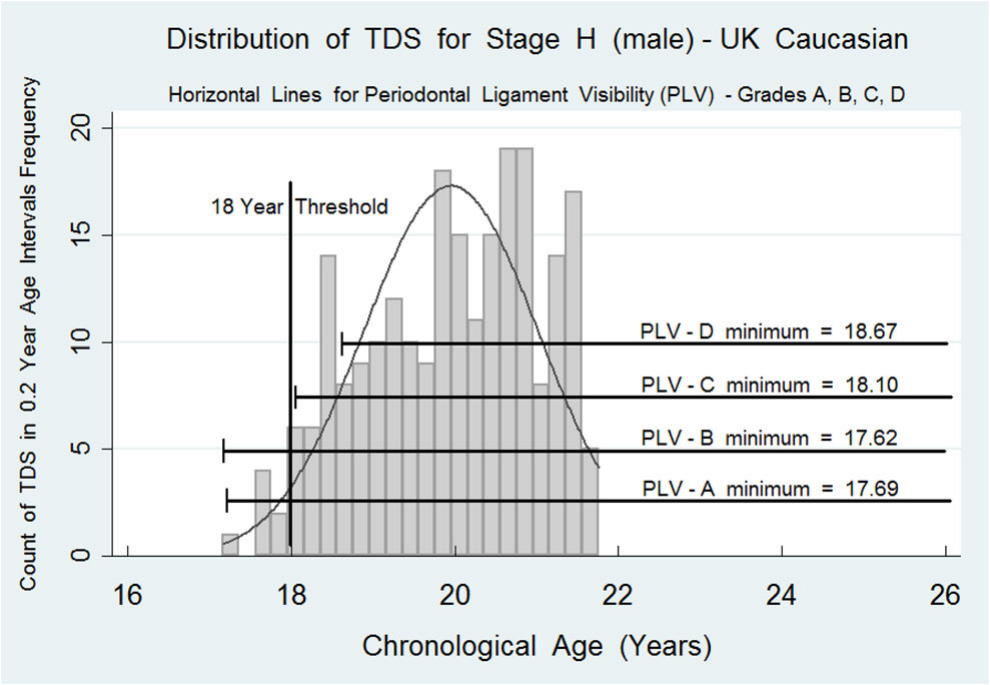
The histograms represent the probability distribution for the ages of attainment for tooth development stage H (the final stage of tooth development) for the lower third molar on the males. The data are censored at approximately 21.6 years for males. Superimposed are the *horizontal data bars* for PLV stages A, B, C, and D. The full summary data for these PLV stages in both females and males are shown in Table 1. The probability distribution curve for LLH f and m, respectively, is present as a background to the *horizontal lines* encompassing the data for PLV-A, PLV-B, PLV-C, and PLV-D. Minimal data has been entered on the graphs to avoid clutter

The probability distribution function for LL8H for females and males with the distribution of the percentile data indicates that for both females and males PLV-C and PLV-D indicates a strong likelihood that the presence of these mandibular maturity markers are confirmation that stage H *and* PLV-C or PLV-D indicate that the subject is over 18 years of age (Figs. 2 and 3). Equally, stage H *and* PLV-A or PLV-B indicate that the subject could be below 18 years.

## Discussion

The use of periodontal ligament visibility is a compelling indication of a subject’s status as a minor (under 18 years) or above the age of majority (over 18 years). This HBGM demonstrates that when making age assessments at the 18-year threshold, it is helpful to be *sure* as to the “below/above” status of the individual for whom age estimation is being conducted [8].

There are a number of issues relating to the methodology of the periodontal ligament visibility categories. The first publication implies a clear pattern of gradual disappearance of periodontal ligament visibility as shown in the schematic drawings in the first publication on this subject [10]. These drawings are reproduced here (Fig. 1). The clear pattern of PLV shown in the schematic diagrams was therefore used as a guide for the pattern recognition of the approximate percentage of the absence of periodontal ligament on the radiographs.

A shortcoming of the original paper [10] is that there was no information on reproducibility of the assessments for the PLV categories. This is a concern as reliable assessment is the key to the establishment of accurate data arrays for each of the categories. Notwithstanding this, the high Kappa scores for within observer agreement and between observer agreement in this study provide strong support for the use of the four categories described in the original paper [10].

An issue of further importance is whether or not the mandibular maturity markers can be applied to ancestral groups other than UK Caucasians, the work from Germany [10] and Portugal [15] support this. The data in this paper are similar to that from Germany and Portugal and lend support to the view that PLV assessments may be applied to other ancestral groups.

The value of the PLV-A, PLV-B, PLV-C, and PLV-D is shown in Figs. 2 and 3 where the data array for each category is overlaid on the probability distribution histogram. The critical feature is the point estimate for the *minimum* value for data array C and also D. In both females and males, the minimum value is higher than the 18-year threshold. This is strong evidence that a subject with complete root development of the lower third molar, stage H in the eight-stage system [3], combined with PLV-C or PLV-D, is above the 18-year threshold. This work confirms that for a definitive assignment of a subject to above the 18-year threshold, it is necessary to identify a mandibular maturity marker such as PLV-C or PLV-D. Only then can a dental surgeon be confident that the subject under investigation has passed and is beyond the 18-year threshold. This paper gives further support to the seminal work on periodontal ligament visibility published in 2010 [10].
